# Supplementary material for: A multi-dimensional analysis of native and non-native academic research articles in twelve disciplines
Source: PLoS One. 2026 Apr 24;21(4):e0346776. doi: 10.1371/journal.pone.0346776 (PMC13108751; doi:10.1371/journal.pone.0346776)
Supplement: S5 Appendix — (PDF) [file pone.0346776.s005.pdf]

Appendix A: Linguistic features selected in this study

Past tense (VBD)
Perfect aspect (PEAS)
Present tense (VPRT)
Place adverbials (PLACE)
Time adverbials (TIME)
First person pronouns (FPP1)
Second person pronouns (SPP2)
Third person pronouns (TPP3)
Pronoun it (PIT)
Demonstrative pronouns (DEMP)
Indefinite pronouns (INPR)
Pro-verb do (PROD)
Direct WH-questions (WHQU)
Nominalizations (NOMZ)
Gerunds (GER)
Total other nouns (NN)
Agentless passives (PASS)
By-passives (BYPA)
Be as main verb (BEMA)
Existential there (EX)
That verb complements (THVC)
That adjective complements (THAC)
WH-clauses (WHCL)
Infinitives (TO)
Present participial clauses (PRESP)
Past participial clauses (PASTP)
Past participial WHIZ deletion relatives (WZPAST)
Present participial WHIZ deletion relatives (WZPRES)
That relative clauses on subject position (TSUB)
That relative clauses on object position (TOBJ)
WH relative clauses on subject position (WHSUB)
WH relative clauses on object position (WHOBJ)
Pied-piping relative clauses (PIRE)
Sentence relatives (SERE)
Causative adverbial subordinators (CAUS)
Concessive adverbial subordinators (CONC)
Conditional adverbial subordinators (COND)
Other adverbial subordinators (OSUB)
Total prepositional phrases (PIN)
Attributive adjectives (JJ)
Predicative adjectives (PRED)
Total adverbs (RB)
Type-token ratio (TTR)
Word length (AWL)

Conjuncts (CONJ)
Downtoners (DWNT)
Hedges (HDG)
Amplifiers (AMP)
Emphatics (EMPH)
Discourse particles (DPAR)
Demonstratives (DEMO)
Possibility modals (POMD)
Necessity modals (NEMD)
Predictive modals (PRMD)
Public verbs (PUBV)
Private verbs (PRIV)
Suasive verbs (SUAV)
Seem|appear (SMP)
Contractions (CONT)
Subordinator that deletion (THATD)
Stranded preposition (STPR)
Split infinitives (SPIN)
Split auxiliaries (SPAU)
Phrasal coordination (PHC)
Independent clause coordination (ANDC)
Synthetic negation (SYNE)
Analytic negation (XX0)
Attitude markers
Boosters
Identity markers
Noun-noun phrase
Reporting verbs
Transition signals
