# Supplementary material for: A multi-dimensional analysis of native and non-native academic research articles in twelve disciplines
Source: PLoS One. 2026 Apr 24;21(4):e0346776. doi: 10.1371/journal.pone.0346776 (PMC13108751; doi:10.1371/journal.pone.0346776)
Supplement: S6 Appendix — (PDF) [file pone.0346776.s006.pdf]

Appendix B: List of abbreviations

AGRI	agriculture
CRC	Chinese researchers corpus
ECON	economics
EDU	education
HIST	history
LIT	literature
MGT SCI	management science
MED	medicine
MDA	multi-dimensional analysis
NAT SCI	natural science
NRC	Native researchers corpus
PHIL	philosophy
ENGR	engineering
