# Supplementary material for: A multi-dimensional analysis of native and non-native academic research articles in twelve disciplines
Source: PLoS One. 2026 Apr 24;21(4):e0346776. doi: 10.1371/journal.pone.0346776 (PMC13108751; doi:10.1371/journal.pone.0346776)
Supplement: Supplementary file 6 [file pone.0346776.s004.doc]

KMO and Bartlett's Test

Kaiser-Meyer-Olkin Measure of Sampling Adequacy.		.974
Bartlett's Test of Sphericity	Approx. Chi-Square	198364.098
	df	2628
	Sig.	.000

Total Variance Explained

	Initial Eigenvalues			Extraction Sums of Squared Loadings			Rotation Sums of Squared Loadings[a]
Factor	Total	% of Variance	Cumulative %	Total	% of Variance	Cumulative %	Total
1	26.290	36.013	36.013	26.015	35.637	35.637	20.659
2	7.299	9.999	46.012	7.025	9.624	45.261	19.605
3	6.716	9.200	55.212	6.432	8.810	54.071	15.613
4	4.915	6.733	61.945	4.634	6.348	60.419	12.471

Pattern Matrix[a]

	Factor			
	1	2	3	4
Attitude markers	.870			
Reporting verbs	.905			
First person pronouns	.793			
Boosters	.813			
Public verbs	.844			
Emphatics	.913			
Pronoun it	.900			
Present tense	.892			
Identitymarkers	.797			
Perfect aspect	.874			
WH-clauses	.826			
Infinitives	.948			
Attributive adjectives	.886			
Subordinator that deletion	.844			
Necessity modals	.726			
Total adverbs	.953			
Total other nouns	-.934			
Nominalizations	-.551			
Total prepositional phrases	-.789			
Conjuncts		.878		
Other adverbial subordinators		.867		
Causative adverbial subordinators		.715		
Past participial WHIZ deletion relatives		.808		
Present participial WHIZ deletion relatives		.826		
Transitional signals		.975		
Pied-piping relative clauses		.815		
Concessive adverbial subordinators		.820		
Conditional adverbial subordinators		.662		
Split auxiliaries		.853		
Phrasal coordination		.877		
Present participial clauses		.905		
Past participial clauses		.862		
BE as main verb		-.826		
Third person pronouns		-.790		
Predicative adjectives		-.779		
Past tense		-.783		
Existential there		-.861		
Demonstrative pronouns		-.814		
Noun_noun phrase			.792	
Possibility modals			.744	
Analytic negation			.617	
Demonstratives			.744	
Hedges			.761	
Downtoners			.704	
By-passives			.805	
Agentless passives			.865	
That adjective complements			-.932	
That verb complements			-.887	
Predicative modals			-.860	
That relative clauses on object position			-.947	
Amplifiers			-.940	
Suasive verbs			-.974	
Type-token ratio				.947
Word length				.840
That relative clauses on subject position				.769
Split infinitives				.628
Independent clause coordination				.932
Sentence relatives				.872
Gerunds				-.704
Private verbs				-.788
Contractions				-.850
WH relative clauses on subject position				-.900
Discourse particles				
Indefinite pronouns				
Place adverbials				
Pro-verb do				
Seem/appear				
Second person pronouns				
Stranded preposition				
Synthetic negation				
Timen adverbials				
WH relative clauses on object position				
Direct WH-questions				

Extraction Method: Principal Axis Factoring.
Rotation Method: Promax with Kaiser Normalization.

a. Rotation converged in 6 iterations.
