# Supplementary material for: A multi-dimensional analysis of native and non-native academic research articles in twelve disciplines
Source: PLoS One. 2026 Apr 24;21(4):e0346776. doi: 10.1371/journal.pone.0346776 (PMC13108751; doi:10.1371/journal.pone.0346776)
Supplement: Attachment — Submitted filename: Resonse to Reviewers.docx [file pone.0346776.s008.docx]

Dear Dr. Ahmed,

We would like to sincerely thank you and the reviewers for your valuable time and effort in evaluating our manuscript, *A multi-dimensional analysis of native and non-native academic research articles in twelve disciplines*, and for providing us with constructive and insightful feedback. We deeply appreciate the thoughtful comments and suggestions which have significantly helped us improve the quality of our work.

We have carefully addressed all the comments and concerns raised by the reviewers, and we have made the necessary revisions to the manuscript accordingly. Below, we provided a point-by-point response to each of the reviewers’ comments, detailing the changes we have made. We hope that these revisions meet your expectations and further strengthen the manuscript. We used red for the revisions made for Reviewer1’s reference, and we used blue for the revisions made for Reviewer2’s reference.

Once again, we are truly grateful for the opportunity to revise our work and for the guidance provided throughout this process. We look forward to your feedback and hope that the revised manuscript is now suitable for publication in *PLOS ONE*.

Sincerely,

Ghayth Al-Shaibani (PhD)

The corresponding author

Point-by-Point Responses to Reviewers’ Comments

| No. | **Comments from reviewer #1** | **Responses to comments** | **Pages on which the revisions were made** |
| --- | --- | --- | --- |
| 1 | The authors should provide more details on how the sample was selected. Was it randomly selected from various disciplines, or were specific criteria used to select the participants? How were "native" and "non-native" researchers defined, and how large were the sample sizes for each group? Clearer explanation of these factors is necessary to ensure that the study's results are generalizable. | We sincerely thank the reviewer for this insightful comment. We have revised this part in the manuscript and highlighted the revisions in red. This research does not involve any human participants; and it uses the method of textual analysis involving academic research articles written by native English researchers and Chinese researchers. The textual analysis of linguistic differences of these research articles is the main research focus. Therefore, the sample selected in this research is research articles in various disciplines.  As for the selection criteria, firstly, the criterion for selecting the disciplines is based on the Chinese National System of Level One Disciplines for Degree Education, namely, agriculture, art, economics, history, law, literature, management science, medicine, natural science, education, philosophy, engineering and military. To make the native English researchers’ corpus and Chinese researchers’ corpus comparable, we selected the same disciplines and same number of the research articles to build the corpora. When we checked native English researchers’ disciplines in the Web of Science (WoS), there was no military discipline. Therefore, the number of final disciplines involved in this study was 12 by excluding military discipline.  Secondly, after deciding the disciplines, for selecting native English researchers’ research articles (RAs), we used Journal Citation Reports (JCR) of the WoS to look for the most influential 10 international Science Citation Index (SCI), Social Science Citation Index (SSCI) and Science Citation Index Expanded (SCIE) journals in each discipline according to the journals’ highest impact factor since the native English researchers’ RAs were set as the norm and then randomly selecting 5 journals from these 10 in each discipline to select RAs (Table 1). In each journal, 20 RAs from 2019-2023 were collected. Thus, the sample size for native English researchers corpus is 1200 articles. As for selecting the research articles for Chinese researchers, simple random sampling was conducted; that is, academic English journals published in China by referring to Bao and Zhang (2018) were randomly selected because it would reflect the Chinese researchers’ average English writing level in this way. Therefore, the sample size for Chinese researchers corpus is 1200 articles as well. In total, the sample size for the corpora is 2400 research articles.  Pertaining to defining the native researchers, we followed Pan (2018)’s approach “the standard for native researchers is basically the authors’ names should be considered native to English-speaking countries as the UK, the USA, Canada, Australia and New Zealand and were affiliated with an institution in a country where English is spoken as the first language” (Pan 2018, 119). | p.11-15. |
| 2 | There is no clear mention of how potential confounding variables, such as the researchers' academic backgrounds, proficiency in English, or cultural differences, were controlled. The authors should consider discussing how they accounted for these potential biases to strengthen the study's validity. | We really appreciate this valuable suggestion. However, we did not consider the variables as researchers' academic backgrounds, proficiency in English, or cultural differences when comparing the native English and non-native researchers’ writing differences because the main focus of this research is to explore how different the academic writing written by Chinese researchers with an average writing level from that of high-quality writing level of native English researchers is. We would like to show the Chinese researchers what the writing style a well-written and high-quality native English research article is, and what difference of theirs is from those well-written ones. So we didn’t control the bias of choosing the same level of proficiency in English and academic research background to make comparisons between native English and Chinese researchers. Instead, we selected the native English research articles from the top journals with highest journal impact factor in each discipline in order to set these native English research articles as a benchmark, but for Chinese researchers’ English research articles we selected the articles from journals with varying impact factors and with average writing level. Furthermore, this is possible because this was done in Cao and Xiao (2013)’s study as well. To control the bias in this research, we used the method of triangulation (of investigators), that is, we assigned three research assistants to collect, clean and analyze the data. We trained these assistants first, and made them completely clear about the rule of selecting journals and research articles, how to clean the texts and use the software to analyze the data. For the quantitative data, we used KMO to test the validity of the data. We added section 3.2.3 to explain the validity and reliability in this study and marked them in red. | p.22-23 |
| 3 | Data Collection Methods: The paper does not specify what kinds of data were collected—was the analysis based on qualitative data (e.g., textual analysis) or quantitative data (e.g., citation counts, journal acceptance rates)? Additionally, the statistical methods used to analyze the data are not outlined. A clear description of the statistical tests performed is essential to assess the rigor of the analysis. | Thank you very much for this valuable comment. We have modified section 3.1 to better present the statistical methods used to analyze the data, and marked in red.  The data collected in this research are published research articles written by the native English and Chinese researchers. To analyze the linguistic features of these research articles, the analysis was based on the qualitative data. We used textual analysis to analyze the articles. The textual analysis was conducted by first extracting the linguistic features suitable for academic writing in the collected research articles, quantifying the frequency of cooccurred linguistic features, then revealing and explaining these cooccurred dimensions after factor analysis. This data collection and data analysis approach was adopted from Biber (1988). Biber pioneered this multi-dimensional analysis approach and then numerous studies followed this approach. The whole data analysis procedure was added in section 3. | p.11-16 |
| 4 | There is no mention of the statistical methods used to test hypotheses or analyze the data. This omission raises concerns about whether the statistical analysis was performed rigorously. I recommend that the authors provide more detailed information on the types of statistical tests used and whether the sample sizes were sufficiently large to ensure meaningful results. Additionally, the manuscript should include more information on how the data were processed and analyzed. | Thank you very much for this valuable feedback. We have revised this section to clarify the statistical methods and how the data were processed and analyzed. All the revisions were marked in red. As for the sample size, we have 2400 articles as a sample which is sufficient for doing this research because in a factor analysis, the data base should include five times as many texts as linguistic features to be analyzed (Gorsuch 1983, p.332). In our study, 62 linguistic features were identified and included, and 310 (62*5) research articles as a sample are enough for this study, while we have sufficient 2400 articles as a sample for this study. | p.11-16 |
| 5 | The manuscript does not mention whether the underlying data supporting the findings will be made publicly available. To enhance the transparency and reproducibility of the study, the authors should make the data available, either in a public repository or as supplementary material. This would allow other researchers to verify the results and build upon this work. | Thank you very much for highlighting this issue. We have uploaded all the underlying data supporting our findings as the supplementary material file. |  |
| 6 | While the conclusions drawn in the manuscript are logical, they need to be more firmly supported by data. The authors mention that non-native researchers face challenges in meeting academic writing standards and publishing in high-impact journals, but the manuscript does not provide specific quantitative or qualitative data to back up these claims. A clearer connection between the data and the conclusions would make the study's findings more robust. | We are grateful for this valuable feedback. We have revised the conclusion section to clarify this point and marked it in red. | p.46-51 |
| 7 | The manuscript is written in standard academic English, and the ideas are generally presented clearly. However, some sentences are overly complex and could be simplified for better readability. I recommend that the authors revise some of the more convoluted passages to improve the overall flow of the paper. | Thank you very much for your suggestion. Our manuscript has been edited by proficient English writer |  |
| 8 | There are no concerns regarding research ethics in this manuscript. The research appears to be conducted in accordance with ethical standards, and there is no indication of any issues with plagiarism or improper citation practices. | Thank you very much for your great feedback. We have already obtained the approval of the ethical application for this study from UCSI university in Malaysia as attached in the supplementary file. |  |
| 9 | The manuscript does not appear to have been previously published or under review elsewhere, based on the information provided. However, I recommend the authors ensure that the paper is not submitted to multiple journals simultaneously and that proper citations and acknowledgements are included. | Thank you very much for your great feedback. Our manuscript has been submitted to PLOS ONE only. For acknowledgements, PLOS ONE required authors should not acknowledge editors and reviewers in the acknowledge section, only to those contribute to the research but not listed as the co-authors. |  |
| No. | **Comments from reviewer #2** | **Responses to comments** |  |
| 1 | Yes/no research questions are not recommended, it is better to re-phrase the third question to be 'To what extent language backgrounds ...?'. | Thank you very much for your good suggestion. We revised third research question and marked it in blue. | p.6 |
| 2 | I suggest having both sections 1 and 2 under one section titled 'Literature Review'. | Thank you very much for your good feedback.  I think you meant section 2 and 3, so we already revised section 2 and 3 under one section 2 titled 'Literature Review' based on your feedback and marked them in blue. | p.6, p.8 |
| 3 | Number 5 in 4.1 should be written as a word. | Thank you very much for your good suggestion. I think you meant “*the most influential 5 international SCI…*” under section 3.1. We revised it and marked it in blue. | p.13 |
| 4 | The ampersand '&' should not be used within the text, only used within the in-text citation. | Thank you very much for your good comment. We changed all the '&' into ‘and’ within the text and marked them in blue. | p.30 |
| 5 | Consistency is required in some places such as having POS or PoS, having MDA or the full words, etc. | Thank you very much for pointing out this issue. We corrected all the POS and MDA and marked them in blue. | p.2,8,11,18 |
| 6 | Is the example in "... and the regular expression of which was written as \sabsolute_JJ, ..." correctly written? | Thank you very much for your good feedback. We checked again the example of \sabsolute_JJ, and the like, all of them were all correct. The format written in this way is a regular expression in computer codes. |  |
| 7 | Figure 1 is to be placed in its correct position within the text so readers can make use of it. The same applies to the other figures. | Thank you very much for your good suggestion. We have followed the journal’s guidelines. The journal guidelines suggest all figures must be submitted separately and only figure captions can be put in where figures should be. |  |
| 8 | I wonder why the researchers do not use 'interactional' and 'informational' instead of positive and negative with regard to Table 3 and what comes after! The word negative gives the impression of having a very low value. To remove this vagueness, it is better to make it clear that both positive and negative are used to help in drawing the line graphs rather than underestimation. | Thank you very much for your feedback. To explain this point, words as 'interactional' and 'informational' are our unique interpretation for each dimension based on the linguistic features extracted after factor analysis; however, positive and negative are used to describe factor loadings. In factor analysis, a loading represents the correlation coefficient (or standardized regression coefficient) between a variable and a factor. The values of loadings typically fall within the range of [-1 and 1]. When a loading is positive, it indicates a positive correlation between the variable and the factor. That is, as the factor score increases, the value of the variable also tends to increase. Conversely, a negative loading implies a negative correlation. In this case, as the factor score rises, the value of the variable tends to decrease. So negative does not imply having a very low value, and it only shows its correlation with this factor. It is *positive* and *negative loading* that distinguished various dimensions and reflected the difference between native English researchers’ and Chinese researchers’ academic writing. |  |
| 9 | Some pedagogical implications can be added to the conclusion section. | Thank you very much for your good suggestion. We have revised the conclusion section and marked the revisions in red because the first reviewer also requested this revision | p.49-50 |
| 10 | Editing is needed to fix some issues. | Thank you very much for your good suggestion. Our manuscript has been edited by proficient English writer. |  |
