# Supplementary material for: A multi-dimensional analysis of native and non-native academic research articles in twelve disciplines
Source: PLoS One. 2026 Apr 24;21(4):e0346776. doi: 10.1371/journal.pone.0346776 (PMC13108751; doi:10.1371/journal.pone.0346776)
Supplement: Attachment — Submitted filename: Resonse_to_Reviewers_auresp_2.docx [file pone.0346776.s009.docx]

Dear Dr. Ahmed,

We would like to sincerely thank you and the reviewers for your valuable time and effort in evaluating our manuscript, *A multi-dimensional analysis of native and non-native academic research articles in twelve disciplines*, and for providing us with constructive feedback. We appreciate the thoughtful comments and suggestions, which have significantly helped us improve the quality of our research.

We have carefully addressed all the comments and concerns raised by the reviewers, and we have made the necessary revisions to the manuscript accordingly. Below, we provide a point-by-point response to each of the reviewers’ comments, detailing the changes we have made. We hope that these revisions meet your expectations and further strengthen the manuscript.

We hope that the revised manuscript is now suitable for publication in *PLOS ONE*.

Sincerely,

The authors

Point-by-Point Responses to Reviewers’ Comments

| No. | **Comments from reviewer #3** | **Responses to comments** |
| --- | --- | --- |
| 1 | Typo in Line 226: "Twentyfull-text articles in each…" | We sincerely thank the reviewer for this careful comment. We have revised this sentence in Section 3.1, line 226, page 13. |
| 2 | The study shows the differences between native authors and Chinese authors. However, one suggestion by the study is: "This finding indicates Chinese researchers should exhibit their authorial stance and interact with the readers with confidence and employ more interactive devices to make their writing coherent and explicit." Why? Can the way native authors promote the influence of articles? Any evidence? | We really appreciate this valuable comment. The reasons for this suggestion can be found in **Section 4.2.** By tagging, counting and analyzing the linguistic features of the collected research articles, we found that, for example, on Dimension One, native English researchers showed a heavy reliance on the use of linguistic features, such as first person pronouns (e.g. *we, our*), reporting verbs (e.g. *require, propose*), adverbs (e.g. *directly, instead, wholly, surprisingly, conceptually*), attributive adjectives (*previous, multiple, simple, significant*) and present tense. These linguistic features create an image of involving the author’s interaction with the readers to highlight the author’s stance explicitly (detailed explanation can be found in **Section 4.2.1**). In contrast, Chinese researchers prefer to use many nouns, nominalizations and prepositions marking the article with dense information, indicating that Chinese researchers avoid involving and interacting with the readers to conceal their authorial identity and opinion, but tend to convey information objectively. Other findings and explanations on Dimension Two, Three and Four can be found **from section 4.2.2 through section 4.4.4**. Therefore, we identified, tagged and counted linguistic features frequencies using Stanford Part of Speech (PoS) tagger and PatCount software. Then we conducted a factor analysis and textual analysis to obtain these findings in our research (pp.17-23).  Native English authors indirectly influence non-native authors. Furthermore, our data on native English researchers are all empirical data collected from research articles published in top international journals and authored by native English researchers. They can be set as a benchmark of high-quality writing (Lines 223-226). Therefore, our findings related to the writing style of native English researchers are not aimed to promote their style, rather it is based on the empirical data we collected from native English researchers’ high-quality research articles which show us the way how most influential articles were written by native English researchers. In other words, we need a benchmark for Chinese researchers to learn from, so that the Chinese researchers can imitate the native English researchers’ writing style to improve their writing. |
| No. | **Comments from reviewer #4** | **Responses to comments** |
| 1 | Line 52: The verb tense and overall language use should be reviewed to ensure consistency with the formal tone expected in academic writing. | Thank you very much for your good suggestion. For this and all following comments on language use, we have already sent our manuscript to Luzhou Jiayi Language Service Company for language editing by professional language editors, and we submitted the language editing certificate for you to check. |
| 2 | Lines 73, 74, 127, 188, 372, 864: The readability of these sections could be improved. Revising sentence structure and ensuring clarity would enhance the overall flow and accessibility of the text. | The response is in number one above. |
| 3 | Abstract Discrepancy: There is an inconsistency between the two versions of the abstract. Notably, the second version omits reference to Nini’s MAT, which is included in the first. The abstracts should be aligned to maintain consistency and accurately represent the study’s scope | Thank you very much for your comment. The first two reviewers requested a revision and thus we revised the whole manuscript including the abstract based on the first-round comments given by the first-round reviewers. |
| 4 | Page 66 (Line 226): Spelling errors, such as “pattens,” should be corrected. A careful proofreading of the manuscript is recommended to eliminate such typographical issues. | Thank you very much for your comment. There is no page 66, but we checked Line 66 and this spelling error was on line 66. We have corrected the spelling errors in the whole manuscript. Besides, we sent our manuscript to Luzhou Jiayi Language Service Company for language editing by professional language editors and submitted the language editing certificate. |
| 5 | Research Question 3: The response to Research Question 3 appears underdeveloped. Its current focus on language of publication is overly restrictive and does not reflect the potential complexity of the issue. A more comprehensive exploration is needed. | Thank you very much for your comment. In fact, the focus of RQ 3 (and even the whole manuscript) is not on the language of publication, but rather the language of writing style differences between native English and Chinese researchers’ academic writing. In other words, there are differences in their academic writing style as each group has a distinctive pattern as reported in our study. We focused on such differences by employing a multi-dimensional model (line 790-794, pp.47). Hence, the answer of RQ3 was explained as how researchers with different language backgrounds (native English researchers and Chinese researchers) write differently on each dimension of our developed model. Through calculating the dimension score of native English researchers’ writing and Chinese researchers’ writing in 12 disciplines on each dimension (Line 423-444, Line 536-560, Line 643-664, Line 719-733), we made a comparison. After that, we used ANOVA to test whether the language background (native English and Chinese) and discipline can influence the dimension score or not. The detailed explanation for RQ3 can be found from Section 4.2.1through 4.2.4. For example, ANOVA tests showed that the main effect of language backgrounds on Dimension 1 was statistically significant, suggesting that Dimension One can differentiate research articles written by native English researchers and Chinese researchers. By doing this, RQ3 (how language backgrounds and disciplines influence the dimension scores of native English and Chinese researchers’ academic writing) was answered. |
| 6 | Line 316: The inclusion of the software download link in the body of the paper is unnecessary. | Thank you very much for your suggestion. We have deleted the link. |
| 7 | Figures and Layout: Figures are not effectively positioned within the text, which disrupts the logical flow and readability. They should be placed adjacent to the relevant discussions to support the argument and maintain textual coherence. | Thank you very much for your comment. However, we are following the journal’s requirement for formatting the submission. Figures must be submitted separately and only figure captions can be put in where figures should be. Kindly take note we are not allowed to breach the journal guidelines. All the figures are placed after the references list based on the submission system. |
| 8 | Clarity of Contribution: The contribution of the paper, particularly the proposed novel MDA model, would benefit from a visual representation. A schematic or diagrammatic illustration would make the model’s structure and innovative aspects more accessible to readers. | Thank you very much for your suggestion. We followed your suggestion, and now there is a diagram as Figure 5 added in the conclusion section in the manuscript. |
| 9 | Strength of Argumentation: The argument that “language background and discipline cannot only account for the variation on dimension scores alone, but also influence the dimension scores altogether” is currently unconvincing. The concept of "language background" is applied and analyzed in a rather narrow way. It is recommended that the authors revisit and refine this argument, potentially in relation to a revised and more robust treatment of Research Question 3, to enhance the theoretical and empirical soundness of their claims. | Thank you very much for your comment. In our study, language background refers to native Chinese language background and native English language background. The whole manuscript only focused on the comparison of language difference between native English researchers and Chinese researchers in academic writing style. Therefore, our argument “language background would influence the dimension score” was obtained from ANOVA test, meaning that native English researchers wrote differently from Chinese researchers, and the difference is significant, i.e. judging from the linguistic features of the research articles, research articles written by native English researchers and Chinese researchers can be differentiated. Anyway, since you advise us to revise this argument, we deleted and revised some parts of this argument from page 49-50. |
