# Supplementary material for: A multi-dimensional analysis of native and non-native academic research articles in twelve disciplines
Source: PLoS One. 2026 Apr 24;21(4):e0346776. doi: 10.1371/journal.pone.0346776 (PMC13108751; doi:10.1371/journal.pone.0346776)
Supplement: Attachment — Submitted filename: Resonse_to_Reviewers_auresp_3.docx [file pone.0346776.s010.docx]

Dear Dr. Ahmed,

We would like to sincerely thank you and the reviewers for your valuable time and effort in evaluating our manuscript, *A multi-dimensional analysis of native and non-native academic research articles in twelve disciplines*, and for providing us with constructive and insightful feedback. We deeply appreciate the thoughtful comments and suggestions which have significantly helped us improve the quality of our work.

We have carefully addressed all the comments and concerns raised by the reviewers, and we have made the necessary revisions to the manuscript accordingly. Below, we provide a point-by-point response to each of the reviewers’ comments, detailing the changes we have made. We hope that these revisions meet your expectations and further strengthen the manuscript.

We look forward to hearing from you soon and hope that the revised manuscript is now suitable for publication in *PLOS ONE*.

Sincerely,

Jiaqi Deng & Ghayth Al-Shaibani

Point-by-Point Responses to Reviewers’ Comments

| No. | **Comments from reviewer #5** | **Responses to comments** |
| --- | --- | --- |
| 1 | I find it unclear what new insights this work contributes to the existing body of literature. | Thank you very much for this valuable feedback. We reanalyzed our data by using a nonparametric approach, an Aligned Rank Transform (ART ANOVA) instead of previous two-way ANOVA because the data fall under non-normal distribution. This yielded new findings on the disciplinary variations, i.e., even for hard sciences that emphasize objectivity and logical rigor, native English researchers exhibit more flexibility by appropriately incorporating authorial identity and engagement with the readers, rather than being informational and objective as it should be. This finding demonstrated native English researchers strategically declared their authorial stance within the disciplinary norms due to their greater writing fluency and more flexible expressions in writing, but the Chinese researchers’ “being informational in writing” tend to be conservative as they follow pre-set disciplinary conventions. While in soft disciplines, the difference is insignificant. Academic writing in soft disciplines permits a diversity of rhetorical styles and modes of expressions. This intrinsic variability may obscure differences induced by various language backgrounds, making statistical differences insignificant.  Another contribution in this research is that we developed a novel MDA model for academic writing in research articles genre and introduced a method of tagging by using PatCount which identifies and codes regular expressions to tag specific linguistic features. This is another contribution because tagging specific linguistic features has been a stumbling block for many corpus linguistics researchers to develop customized MDA model based on their research purpose.  The detailed revised discussion and conclusion sections can be found on pages 28-30, 34-42, 46-47, 53, 57-61. |
| 2 | For instance, the method of determining an author’s first language is problematic. Without directly contacting authors, it is virtually impossible to verify whether a given article was written by an L1 English speaker. Relying on names and institutional affiliations as proxies for “native” identity risks serious misclassification; for example, a Chinese author may have lived and studied in an English speaking country for an extended period before returning to China, while many L2 scholars work at English-medium institutions. | Thank you for raising this important point. We fully agree with the reviewer.  We adopted this method for the following reasons: First, directly contacting all authors to confirm their L1 identity was not feasible for a study of this scale with 2400 articles. Second, the use of the first author’s name and institutional affiliation as proxy indicators is a widely adopted method in Applied Linguistics and English for Academic Purposes (EAP) research for conducting large-scale analyses. The following studies adopted the author’s name and the nation of institution to determine native researchers. For example, Abdi and Farrokhi (2015) compared the use and functions of first-person pronouns in L1 and L2 research articles of Applied Linguistics (AL), Mechanical Engineering (ME), and Medicine (MED) whereby “Articles were judged to be L1 or L2 considering the authors’ names and affiliations” (p.158). In another research, “verification about author nativeness was not ensured by contacting them. Authors’ status of nationality was presumed based on their names or nationalities (Yagizi & Demir, 2015, p.15). In Pan (2018)’s research, “L1 research articles were selected from prestigious international journals (measured by impact factors) whose authors had a first and last name that can be considered native to English-speaking countries and were affiliated with an institution in a country where English is spoken as the first language” (p. 119). Kareema and Hakmal (2023) collected abstracts written by Sri Lankan scholars and Western scholars related to the English field randomly stating that “Both journals are available online and the articles were all checked in terms of the author's nationality” (p. 323). In Cao and Xiao’s (2013) research published on *Corpora*, they built the corpora of English abstracts written by native English and native Chinese writers from twelve academic disciplines. However, they only considered the most prestigious journals in each discipline as reflected by their impact factors to select native English speakers’ abstracts, and also to select journals with varying impact factors for the Chinese speakers’ abstracts.  We recognize that this classification cannot fully capture the complexity of scholarly identity, but it is widely used in our field. Meanwhile, in our study, we used a double-safeguard method to maximize the chances of the included researchers in *native researchers corpus* are native researchers. This double-safeguard way was explained in detail in the manuscript on pages 14-15 as well. First, the nation of the institution affiliated with which the paper is published was determined. Only institutional affiliation belongs to nations in the inner circle of English-speaking countries, specifically the United States, United Kingdom, Canada, Australia, and New Zealand were included. Papers with which the nationality of the institution cannot be determined or not belong to these five English-speaking nations were discarded. Then we determined whether the first author’s nationality was matched with that of their publishing institution, by using a name origin database. If the ethnic origin of the first author’s surname was consistent with the nationality of the publishing institution, the author was regarded as originating from that nation.  Papers that did not meet this criterion were excluded from the final corpus. Referring to the reviewer’s comment here “many L2 scholars work at English-medium institutions”, we first checked whether the English-medium institutions are in the United States, United Kingdom, Canada, Australia, or New Zealand, if not, they are excluded from this research; if yes, we then checked the L2 scholar’s surname. We know that L2 scholars have unique surnames different from native English speakers, for example, Chinese L2, Japanese L2, German L2, Russia L2, Arabic L2, Portuguese L2 and so on. Only when the nationality of the surname of the first author belongs to is the same as where the institution is located, we regarded the research as done by native researchers. In this way, we may have excluded research articles written by native researchers, but we insured that the researchers included in our research are maximally native.  The reviewer also mentioned here “a Chinese author may have lived and studied in an English speaking country for an extended period before returning to China”, yes, we did not set the standard to select Chinese scholars because in this study we did not consider Chinese scholar’s English proficiency as a variable, we explained this on page 15, we collected all English levels’ Chinese scholars in order to reflect the Chinese researchers’ average English writing level.    **Cited sources list**  Abdi, J., & Farrokhi, F. (2015). Investigating the projection of authorial identity through first person pronouns in L1 and L2 English research articles. *International Journal of Language and Literature*, *3*(1), 156-168. (Scopus Q1)  Cao, Y., & Xiao, R. (2013). A multi-dimensional contrastive study of English abstracts by native and non-native writers. *Corpora*, *8*(2), 209-234. (ESCI Q3)  Kareema, M. I. F., & Hakmal, M. H. M. (2023). A Genre Analysis of Abstracts Written by Sri Lankan and Western Academics on Social Science Discipline. *KALAM International Research Journal*, 16(1), 319-330.  Pan, F. (2018). A multidimensional analysis of L1–L2 differences across three advanced levels. *Southern African Linguistics and Applied Language Studies*, *36*(2), 117-131. (SSCI Q4)  Yagiz, O., & Demir, C. (2015). A comparative study of boosting in academic texts: A contrastive rhetoric. *International Journal of English Linguistics*, *5*(4), 12. |
| 3 | In addition, the study’s corpus design raises concerns. Corpus-based research typically faces challenges in controlling the number and distribution of research articles, and the criteria here are not sufficiently justified. | Thank you very much for this valuable comment. We noticed this and justified it in our manuscript as explained on page 16. First, for the number of research articles selected for the corpus design, we referred to Gorsuch (1983) whereby in a factor analysis, the database should include five times as many texts as linguistic features to be analyzed (p.332). In our study, 62 linguistic features were ultimately identified and included. Thus, 310 research articles are enough as far as the number of research articles for this study’s corpus design is concerned. In our study, we have collected 2, 400 articles as a sample for our corpus design which is sufficient for corpus-based research (Gorsuch, 1983).  As for the distribution of research articles, we aim to distinguish the disciplinary variation among the selected research articles, so we determined 12 disciplines for the corpus. The criteria for selecting the disciplines followed the *Chinese National System of Level One Disciplines for Degree Education*, and the selection and distribution of the research articles were fully explained on page 13 through page 16. |
| 4 | With respect to Research Question 3, although ANOVA tests are reported, the discussion is underdeveloped. The implications of significant interactions are only briefly noted and not adequately theorized in terms of disciplinary writing practices and cultures. | Thank you very much for this valuable feedback. To revise the discussion section for RQ3 with theoretical and practical implications, we re-analyzed our data. Due to the non-normal distribution of the data, a nonparametric approach, an Aligned Rank Transform (ART ANOVA) was conducted to replace the previous Two-way ANOVA. Then we reanalyzed and revised the results and re-wrote the discussion section. This was done to yield deeper and insightful implications in the discussion, especially on Dimension 1, and conclusion sections. Most pages of extensive discussion of Dimension 1 were provided because only on Dimension 1, the main effect of discipline on the dimension score was statistically significant based on our new ART ANOVA test. Then a focused and deeper discussion on the disciplinary differences was done according to the reviewers’ comments. The revisions made can be found on pages 28-30, 34-42, 46-47, 53, 57-61. |
