# Supplementary material for: A multi-dimensional analysis of native and non-native academic research articles in twelve disciplines
Source: PLoS One. 2026 Apr 24;21(4):e0346776. doi: 10.1371/journal.pone.0346776 (PMC13108751; doi:10.1371/journal.pone.0346776)
Supplement: Attachment — Submitted filename: Resonse to Reviewers & Editor.docx [file pone.0346776.s011.docx]

Dear Editor,

We would like to sincerely thank you for your valuable time and effort in evaluating our manuscript, *A multi-dimensional analysis of native and non-native academic research articles in twelve disciplines*, and for providing us with constructive and insightful feedback. We deeply appreciate the thoughtful comments and suggestions which have significantly helped us improve the quality of our work.

We have carefully addressed all the comments and concerns raised by you, and we have made the necessary revisions to the manuscript accordingly. Below, we provide a point-by-point response to the comments, detailing the changes we have made. We hope that these revisions meet your expectations and further strengthen the manuscript.

We look forward to hearing from you soon and hope that the revised manuscript is now suitable for publication in *PLOS ONE*.

Point-by-Point Responses to Reviewers’ Comments

| No. | **Comments from reviewer #1** | **Responses to comments** |
| --- | --- | --- |
| 1 | First, the novelty of the study’s contributions could be more explicitly linked to gaps in the existing literature to highlight its unique insights. | Thank you very much for this valuable feedback. We have rewritten the Conclusion (page 58-63) and added a new opening paragraph to the Conclusion that explicitly positions our contributions against MDA limitations: lack of academic-genre frameworks and effective method of tagging linguistic features for specific domains, and insufficient disciplinary nuances. We now state: “This study addresses these gaps by: (1) selecting 62 academic-specific linguistic features; (2) using PatCount for automated tagging; and (3) deriving four novel dimensions for academic writing across 12 disciplines”. |
| 2 | Second, the issue of misclassification regarding scholars with high English proficiency has been addressed, but further elaboration on how this issue was dealt with could make the methodology more robust. | Thank you for your comment. To clarify our classification approach, which builds directly on our prior response and established field practices, in the revised manuscript (Methods section, pages 14 &15), we have elaborated our double-safeguard method: (1) restricting to institutions in core English-speaking countries (US, UK, Canada, Australia, New Zealand), excluding non-core or undetermined affiliations; and (2) verifying first-author surname origin matches the institution’s country via name database, specifically filtering out L2-associated surnames (e.g., Chinese or Japanese). This addresses Round 3 reviewer’s concerns about L2 scholars at English-medium institutions (by country restriction first) and extended immersion (acknowledged as a proxy limit). For Chinese authors, we intentionally sampled across proficiency levels (as noted on page 15) to capture average writing patterns because proficiency is not a variable. Therefore, no changes to the results are needed as sensitivity to misclassification is minimal given the sample size.    To elaborate further, this double-safeguard proxy method virtually eliminates misclassification of L2 authors as L1 by requiring both institutional location in core English-speaking countries and surname origin matching that country. While rare edge cases exist (e.g., naturalized citizens who legally changed their ethnic surnames to Western names), such instances are exceptional in academic publishing and unlikely to affect group-level patterns in a corpus of 2,400 articles. However, we have added a Limitations section (pages 57 & 58) to explain this classification. |
| 3 | Additionally, while the corpus design has been justified, more clarity is needed regarding the representativeness of the sample, particularly in terms of the diversity within disciplines. Expanding on how the corpus accounts for variations within each discipline would help strengthen the generalizability of the findings. | We thank you for drawing attention to the issue of disciplinary diversity. In the revised manuscript, we clarified that the corpus was constructed based on the Chinese National System of Level One Disciplines for Degree Education which includes 12 broad disciplines (agriculture, art, economics, history, law, literature, management science, medicine, natural science, education, philosophy, and engineering). Within each discipline, we sampled research articles without further subdiscipline classification, as our focus was on broad disciplinary patterns rather than highly specialized subfields. We now explicitly acknowledge this as a limitation in Section 4 (pages 58), noting that our findings should be interpreted as generalizing average tendencies at the level of major disciplines, and we suggest that future research can build larger, subdiscipline-stratified corpora to examine finer-grained variation. |
| 4 | Moreover, a more balanced approach to comparing the writing styles of native and non-native researchers would improve the objectivity and comprehensiveness of the study. While the author highlights areas where Chinese researchers can improve, such as authorial stance and interaction with readers, it would be important to also consider the strengths of native English writers. For example, Chinese scholars often excel at creating concise and information-dense texts, which could offer valuable lessons for English-native researchers. By presenting a more reciprocal view, where both groups can learn from each other, this study would offer a more comprehensive and culturally inclusive perspective on academic writing practices. | Thank you very much for such an insightful suggestion. We revised our whole manuscript and offered a more reciprocal view. We considered the strengths of Chinese researchers for the four dimensions and revised our Dimension 1 on pages 34 & 35, Dimension 2 on page 47, Dimension 3 on page 52, Dimension 4 on page 57. We also revised our abstract and our conclusion on page 61. The revised texts are below:  Dimension 1 (pages 34 & 35): While native researchers excel at authorial engagement and elaboration, Chinese researchers demonstrate strengths in information density and conciseness, efficiently packing content with minimal redundancy, a style valued in fast-paced, technical fields as engineering. This aligns with EAP research showing L2 writers’ precision can model concise argumentation for L1 peers. Thus, both groups offer mutual lessons of natives in interactivity and Chinese in streamlined reporting.  Dimension 2 (page 47): Dimension 2 differentiated native researchers’ interactive argumentation from Chinese researchers’ static descriptive style. This reflects proficiency differences that natives chain ideas linearly with complex connectors, while Chinese EFL writers favor direct *A is B* structures due to vocabulary and syntax constraints. Yet this L2 simplicity confers strengths in clarity and conciseness, avoiding native overelaboration that can obscure meaning. Such straightforwardness enhances readability in disciplines with heavy jargons.  Dimension 3 (page 52): Chinese impersonality offers strengths in objectivity and humility, aligning with disciplinary conventions that prioritize collective knowledge over individual voice. This restrained writing style models cultural sensitivity for natives, who risk perceived bias through overt self-positioning.  Dimension 4 (page 57): Dimension 4 contrasted native researchers’ explicit elaborating style with Chinese researchers’ simplified reporting style. Natives excelled in elaboration due to superior proficiency. Chinese simplicity provides strengths in efficiency and readability, prioritizing research content over linguistic display. This reader-centered writing style models concise impact for natives prone to overelaboration.  Conclusion: In addition to the above-mentioned key findings from different language backgrounds and disciplines, this research also contributed to enriching the existing MDA research by providing a newly complementary perspective. Unlike previous studies stressing the differences, this research innovatively emphasized the mutual strengths from these explored differences. This balanced comparison reveals mutual strengths of Chinese conciseness (Dimensions 1 & 4) model clarity and efficiency for natives prone to verbosity; native involvement and interaction (Dimensions 1 & 4) guides Chinese toward engagement. Chinese impersonality (Dimension 3) offers objectivity where natives risk bias; native logic (Dimension 2) complements L2 simplicity. |
